# Comparison of the Postoperative Outcomes of the Mini-Flap Bilateral Axillo-Breast Approach (BABA) and Conventional BABA Robot-Assisted Thyroidectomy

**DOI:** 10.3390/jcm11164894

**Published:** 2022-08-20

**Authors:** Ik Beom Shin, Dong Sik Bae

**Affiliations:** Department of Surgery, Haeundae Paik Hospital, Inje University College of Medicine, Busan 48108, Korea

**Keywords:** bilateral axillo-breast approach (BABA), robot-assisted thyroidectomy, minimally invasive surgery, minimally invasive thyroidectomy, mini-flap

## Abstract

The bilateral axillo-breast approach (BABA) for robot-assisted thyroidectomy has some advantages over other minimally invasive thyroidectomies. However, some people do not consider this as a minimally invasive thyroidectomy because of the wider surgical skin flap. Thus, we devised mini-flap BABA robot-assisted thyroidectomy and analyzed the postoperative outcomes. The clinical records of 44 patients undergoing BABA robot-assisted thyroidectomy using a conventional flap or mini-flap were evaluated retrospectively. There were no significant group differences in clinicopathological characteristics. The operating and flap making times were shorter in the mini-flap group (206.18 ± 31.09 vs. 178.90 ± 34.43 min, *p =* 0.009; 38.85 ± 2.73 vs. 32.21 ± 8.62 min, *p =* 0.003, respectively). The total drainage amount was smaller in the mini-flap group (196.57 ± 81.40 vs. 150.74 ± 40.80 mL, *p =* 0.027). The numeric rating scale score and number of analgesics were lower at 2 h postoperatively in the mini-flap group (5.52 ± 0.87 vs. 4.57 ± 1.31, *p =* 0.006; 0.95 ± 0.22 vs. 0.65 ± 0.49, *p =* 0.012, respectively). There was no significant group difference in immediate oncological outcomes (*p =* 1.000). Mini-flap BABA robot-assisted thyroidectomy minimized the surgical flap and improved surgical outcomes. Therefore, it is a form of minimally invasive thyroidectomy. However, long-term follow-up of oncological outcomes is needed.

## 1. Introduction

Since the concept of minimally invasive surgery was first introduced [1], various surgical methods have been used for thyroidectomy [2]. Minimally invasive thyroid surgery is classified according to the access site [3] as cervical (shorter incision length but remains in the neck) or extracervical (chest, breast, axillary, and transoral). Minimally invasive thyroidectomy enhances cosmetic satisfaction and reduces postoperative pain, hospital stay, recovery time, and social costs. It also improves the quality of life of patients after thyroidectomy [4,5]. Moreover, because most patients who undergo thyroidectomy are young women, reducing the anterior neck scar using minimally invasive thyroidectomy may increase patient satisfaction [6].

The bilateral axillo-breast approach (BABA) for thyroidectomy has some advantages over other extracervical approaches, including cosmetic satisfaction and a provision of a symmetrical view of both thyroid lobes for the surgeon (which allows the same dissection methods used for open thyroidectomy to be applied, as well as central and lateral compartment dissection [7]). BABA robot-assisted thyroidectomy using the da Vinci surgical system has advantages over endoscopic procedures [8]. It provides a magnified three-dimensional operating field and uses motion filtering and endo-wrist technology to improve ergonomics. BABA robot-assisted thyroidectomy combines the advantages of minimally invasive thyroidectomy and robot-assisted thyroidectomy.

However, BABA robot-assisted thyroidectomy requires a wider surgical skin flap compared to other types of minimally invasive thyroidectomy and conventional open thyroidectomy. Consequently, there is more pain and sensory impairment in the anterior chest and neck [9,10]. Because of these disadvantages, BABA robot-assisted thyroidectomy is not universally considered to be a form of minimally invasive thyroidectomy [11].

We devised the mini-flap BABA robot-assisted thyroidectomy to compensate for these disadvantages. Here, we report the surgical results for 44 cases treated in our institution compared to the postoperative outcomes between mini-flap BABA robot-assisted thyroidectomy and conventional BABA robot-assisted thyroidectomy. We demonstrate that mini-flap BABA robot-assisted thyroidectomy is, in fact, a form of minimally invasive thyroidectomy.

## 2. Materials and Methods

### 2.1. Patients

The clinical records of 44 patients who underwent BABA robot-assisted thyroidectomy using a conventional flap or mini-flap in the Department of Surgery of Haeundae Paik Hospital and Inje University College of Medicine (Busan, Korea) between January 2021 and February 2022 were prospectively collected and retrospectively analyzed. Of the 44 patients, 21 had conventional flaps, and 23 had mini-flaps. All patients underwent fine-needle aspiration (FNA) before surgery; those of Bethesda category ≥ 4 underwent surgery. The inclusion criteria were the same as those used for robotic thyroidectomy in our hospital at that time; there were no additional exclusion criteria. The inclusion criteria were tumor size ≤ 4 cm, even if a clinically suspicious extrathyroidal extension (ETE; cT3) was apparent; no lateral lymph node metastasis (cN1a); no other organ invasion; no distant metastatic disease (cM0). No case required conversion to open thyroidectomy. This study was approved by the Institutional Review Board of Inje University Haeundae Paik Hospital (Approval no. 2022-04-036).

### 2.2. Surgical Procedure

Conventional flap BABA robot-assisted thyroidectomy has been described previously [12]. Two superomedial circumareolar incision sites are demarcated, along with two axillary skin incision sites on existing skin folds, and four oblique lines are drawn from each of these sites to the cricoid cartilage (in the midline). The superior boundary of the flap extends to the upper border of the thyroid cartilage; the inferior boundary extends to two fingers below the clavicle. The lateral boundary is defined by a line drawn from the thyroid cartilage to the point where the line drawn from each axilla meets the upper border of the clavicle.

**Figure 1:**
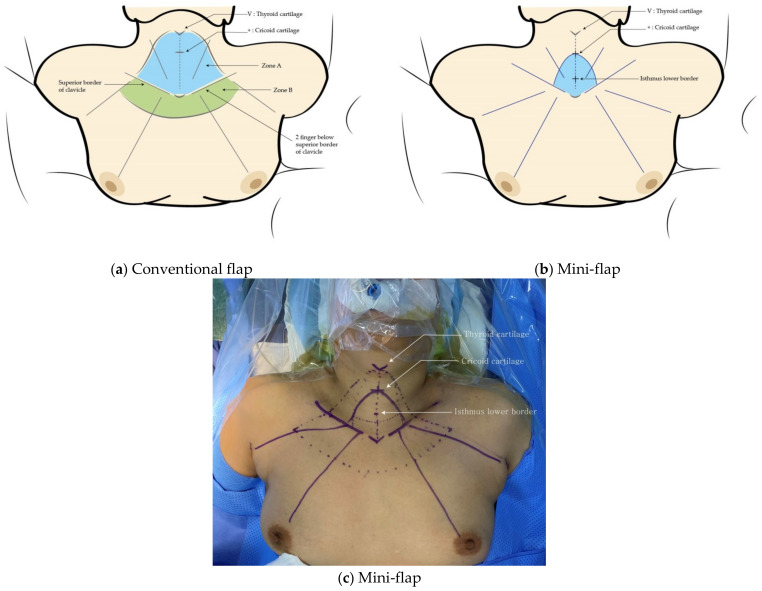
Guidelines for (**a**) conventional BABA robot-assisted thyroidectomy and (**b**,**c**) mini-flap BABA robot-assisted thyroidectomy.

**Figure 2:**
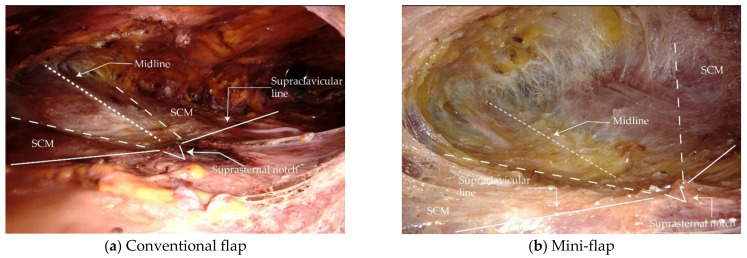

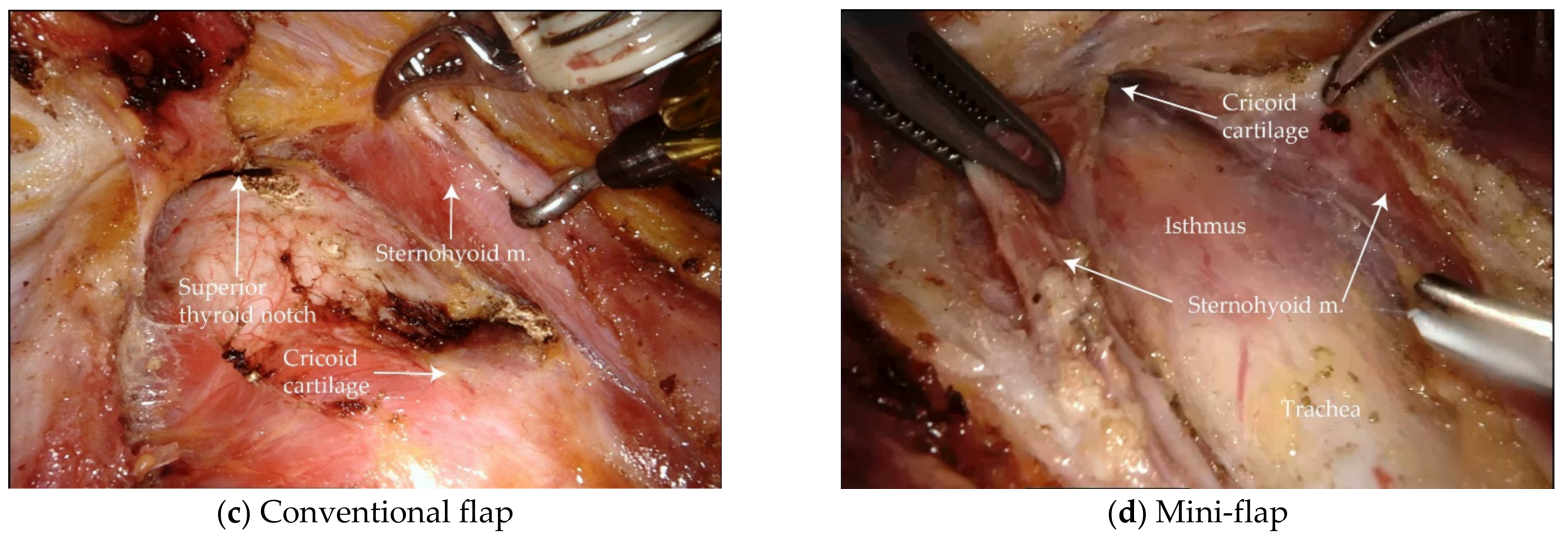
Endoscopic view after making the flaps. (**a**,**c**) Conventional and (**b**,**d**) mini-flap. SCM: sternocleidomastoid muscle.

The mini-flap has different boundaries. The superior boundary extends to the upper border of the cricoid cartilage, while the inferior boundary extends to the upper borders of the clavicle and sternum. The lateral boundary is defined by a line drawn from the cricoid cartilage to the point where the line drawn from each axilla meets the lower border of the isthmus (Figure 1 and Figure 2).

The operative field is demarcated via hydrodissection.

### 2.3. Operative and Postoperative Outcome Measurements

Clinical (amount of drainage, length of hospital stay, operating time, flap making time, estimated blood loss, postoperative pain [numeric rating scale and number of analgesics]), surgical (pathology results and immediate postoperative complications, i.e., hypoparathyroidism and recurrent laryngeal nerve [RLN] injury), and oncological (response to therapy; dynamic risk stratification) data were collected via clinical chart review. The response to therapy was assessed as proposed by Tuttle et al. The serum thyroglobulin levels and imaging results obtained during follow-up were used to evaluate the treatment response (excellent, indeterminate, biochemically incomplete, or structurally incomplete). The length of hospital stay was measured from the day of admission to the day of discharge. All patients underwent thyroidectomy on the day after admission. The operating time was from demarcating the operative field to skin incision closure. The flap-making time was from the injection of dilute epinephrine solution to the start of robot docking. Postoperative pain intensity, quantified using a numeric rating scale that ranged from 0 (no pain) to 10 (worst pain imaginable), was assessed at 2, 24, 48, and 72 h after surgery. The number of analgesics was evaluated at 0–2, 2–24, 24–48, and 48–72 h after surgery. When the numeric rating scale score was ≥4 points or a patient requested an analgesic, intravenous ketorolac (30 mg) was given. Tumor size was provided by the longest tumor diameter. Malignant tumors were staged (T or N stage) following the American Joint Committee on Cancer (AJCC) 8th edition staging system. Postoperative hypoparathyroidism was defined as a low intact parathyroid hormone (iPTH) level (<15 pg/mL) with hypocalcemic symptoms, including a tingling sensation, numbness, and tetany of the hands, feet, or perioral area. Hypocalcemic signs included Chvostek’s and Trousseau’s signs. The iPTH level was measured at 8 a.m. on postoperative days 1–3. RLN injury was defined as loss of vocal cord mobility, which was assessed by rigid laryngoscopy (preoperatively and at the first visit after discharge) (approximately 10 days post-surgery. Transient hypoparathyroidism and postoperative vocal cord palsy resolved within 6 months. If such conditions persisted after 6 months, they were considered permanent. We evaluated oncological outcomes by reference to the therapy category of the dynamic risk stratification [13,14].

### 2.4. Statistical Analysis

The clinical, surgical, and oncological characteristics of the two groups were compared using the *χ*^2^ test or Fisher’s exact test for continuous data and the independent *t*-test or Mann–Whitney *U*-test for categorical data. *p*-values < 0.05 were considered significant. All statistical analyses were performed using SPSS software (ver. 25.0; IBM Corp., Armonk, NY, USA).

## 3. Results

### 3.1. Demographics and Pathological Outcomes

Clinicopathological characteristics (age, sex, body mass index, the extent of surgery, and whether central node dissection was performed) did not differ between the conventional and mini-flap groups (Table 1). The mean tumor size was 0.88 ± 0.60 cm in the conventional group and 1.21 ± 0.85 cm in the mini-flap group (*p* = 0.149). We retrieved more central lymph nodes from the conventional group (6.81 ± 4.39 vs. 4.45 ± 3.20; *p* = 0.050). The number of metastatic lymph nodes did not differ significantly between the groups (1.38 ± 2.58 vs. 0.91 ± 1.38; *p* = 0.455). However, thyroiditis prevalence showed a significant group difference (*p* = 0.042). The most common pathological diagnosis in both groups was papillary thyroid cancer (81.0% vs. 73.9%; *p* = 1.000). The pathological T (*p* = 1.000), N (*p* = 0.845), and AJCC (8th revision) (*p* = 1.000) stage proportions did not differ significantly between the groups (Table 1).

### 3.2. Clinical Outcomes

Table 2 summarize the clinical outcomes. The mean operating (206.18 ± 31.9 vs. 178.90 ± 34.43 min; *p* = 0.009) and flap-making (38.85 ± 2.73 vs. 32.21 ± 8.62 min; *p* = 0.003) times were shorter in the mini-flap group. There were no significant differences between groups in estimated blood loss or hospital stay. The mean total drainage amount was significantly smaller in the mini-flap group (196.57 ± 81.40 vs. 150.74 ± 40.80 mL; *p* = 0.027). Table 3 list the pain parameters. The numeric rating scale (*p* = 0.006) and the number of analgesics (*p* = 0.012) at 2 h postoperatively were significantly lower in the mini-flap group.

### 3.3. Surgical Outcomes

Table 4 summarize the clinical outcomes. The lowest postoperative iPTH levels were 39.98 ± 17.89 pg/mL in the conventional group and 35.79 ± 14.15 pg/mL in the mini-flap group (*p* = 0.457). No postoperative permanent hypoparathyroidism or vocal cord palsy was observed in any patient. There were no postoperative wound complications, including bleeding, infection, or seroma, in either group. Additionally, no safety issues arose during surgery, such as uncontrolled bleeding or permanent nerve damage, hospitalization, or adverse events noted in the outpatient clinic.

### 3.4. Oncological Outcomes

We classified all oncological outcomes except those identified pathologically as benign diseases, including follicular adenoma and Hürthle cell adenoma (Table 5). The response to therapy did not differ significantly between the conventional and mini-flap groups (excellent, 94.1% vs. 83.3%; indeterminate, 5.9% vs. 11.1%, *p* = 1.000).

## 4. Discussions

Various extracervical approaches are used for robot-assisted thyroidectomy in South Korea [15]; BABA robot-assisted thyroidectomy is one of the most widely used approaches [16]. BABA robot-assisted thyroidectomy has many advantages. First, it provides a symmetrical surgical view of important anatomical landmarks, enabling total thyroidectomy while preserving RLN and parathyroid gland [17,18,19]. Second, it provides a surgical view similar to that of conventional open thyroidectomy, so the learning curve is less steep for BABA robot-assisted thyroidectomy than for other methods [20]. Third, it prevents instrument collisions and interference because of the significant distance between ports [21]. Fourth, the recovery times for parathyroid and RLN function are shorter than with open thyroidectomy, while the complication rate (RLN injury and hypoparathyroidism) and surgical completeness do not differ significantly [22,23]. Fifth, restricted areas at the corners of the surgical space can be secured using an articulated robot arm, even without CO_2_ insufflation, as described previously [24].

Despite these advantages, one study stated that BABA robot-assisted thyroidectomy is not a form of minimally invasive thyroidectomy because a larger surgical skin flap is needed than for open thyroidectomy, and sensory impairment and postoperative pain arise in the anterior chest [25]. They argued that BABA robot-assisted thyroidectomy is a form of remote-access surgery rather than being minimally invasive thyroidectomy and that minimally invasive surgery should not be defined based only on the length of the incision or absence of a neck scar. Instead, other criteria such as the invasiveness of all structures dissected during the operation, type of anesthesia, operation duration, postoperative pain, complications, and long-term outcomes should be considered. In conclusion, they argued that minimally invasive thyroidectomy and remote-access thyroidectomy are distinct concepts and that extracervical approaches (including BABA robot-assisted thyroidectomy) should be classified as remote-access thyroidectomy [26,27].

Therefore, we developed the mini-flap BABA robot-assisted thyroidectomy, which reduces the flap area more than both conventional BABA robot-assisted thyroidectomy and conventional open thyroidectomy. Using ImageJ software (ver. 1.53k; National Institutes of Health, Bethesda, MD, USA), the mean flap areas were compared between conventional BABA robot-assisted thyroidectomy and open thyroidectomy. In a conventional open thyroidectomy, the incision is 5 cm long. The mean area of mini-flap BABA robot-assisted thyroidectomy was reduced by 28% compared to conventional open thyroidectomy and by 54% compared to conventional BABA robot-assisted thyroidectomy.

In this study, the mini-flap method reduced the flap-making and operating times compared to conventional methods. In addition, the total amount of drainage was lower in the mini-flap group. The immediate postoperative numeric rating scale scores and numbers of analgesics were also significantly lower in the mini-flap group. Although there was no significant group difference, the hospital stay was shorter in the mini-flap group. The mini-flap also reduced the amount of solution required for hydrodissection. We expect that, as pain intensity is reduced, patient quality of life will improve. Moreover, as the flap area decreases, the trauma associated with surgical exposure should be less severe compared to conventional open thyroidectomy [28]. Consequently, mini-flap BABA robot-assisted thyroidectomy meets the definition of minimally invasive thyroidectomy.

A few technical difficulties associated with mini-flap BABA robot-assisted thyroidectomy should be mentioned. In conventional BABA robot-assisted thyroidectomy, it is easy to change instruments and identify anatomical landmarks because of the wide surgical field. However, in mini-flap BABA robot-assisted thyroidectomy, the surgical field is small, and the distance from the port is long, making it difficult to place the specimen into an endoplastic bag and remove it via the axillary port. We overcame this using an articulated robot arm, placing the endoplastic bag as far down as possible to secure sufficient space, and shortening the axillary port length by compressing the overlying skin. It is sometimes difficult to replace the robot arm because the port insertion length is relatively longer, while the flap size is smaller than those of the conventional method. Thus, delicate maneuvering by an assistant is necessary, and tissue in front of the trocar should be checked with endoscopic forceps before docking the robot arm. It is also difficult to maintain a stable field of view. In conventional BABA robot-assisted thyroidectomy, surgeons obtain a good surgical view by creating “Zone B” (Figure 1). The robot arm should be docked while maintaining the proper angle so that it can be lifted [29,30]. The surgical field is secured by pushing the flap away using an articulated robot arm [24].

Our study had some limitations. First, the operations were carried out by two endocrine surgeons, so there may have been inter-surgeon variability. However, both surgeons were trained in the same institution and had performed more than 100 robotic surgeries over the previous decade. Second, the patient cohort was too small to achieve high statistical power, and the study used a retrospective design. Additional large, prospective, randomized studies are needed. Finally, we analyzed only short-term outcomes; long-term studies are needed to analyze oncological outcomes.

## 5. Conclusions

The new mini-flap BABA robot-assisted thyroidectomy, implemented using the da Vinci robot system, minimizes the surgical flap compared with open thyroidectomy and improves surgical outcomes by reducing the amount of drainage, shortening the hospital stay and reducing the trauma associated with surgical exposure. Therefore, mini-flap BABA robot-assisted thyroidectomy can be classified as minimally invasive thyroidectomy. However, long-term follow-up studies are needed. A prospective randomized controlled trial is in progress at our hospital (Clinicaltrials.gov ID: NCT05216718).

## Figures and Tables

**Table 1:** Clinicopathological characteristics of the patients.

		Group		
	Overall (*n* = 44)	Conventional (*n* = 21)	Mini-Flap (*n* = 23)	*p*-Value
Age, years	40.07 ± 12.01	41.19 ± 12.04	39.04 ± 12.33	0.721
Sex				1.000
Male	5 (11.4%)	2 (9.5%)	3 (13.0%)	
Female	39 (88.6%)	19 (90.5%)	20 (87.0%)	
BMI (kg/m^2^)	24.11 ± 4.22	24.07 ± 2.88	24.15 ± 5.23	0.951
Extent of surgery				1.000
Total thyroidectomy	7 (15.9%)	3 (14.3%)	4 (17.4%)	
Unilateral thyroidectomy	37 (84.1%)	18 (85.7%)	19 (82.6%)	
CND				0.709
Not performed	7 (15.9%)	2 (9.5%)	5 (21.7%)	
Unilateral	36 (81.8%)	18 (85.7%)	18 (78.3%)	
Bilateral	1 (2.3%)	1 (4.8%)	0	
Nodes retrieved	5.60 ± 3.97	6.81 ± 4.39	4.45 ± 3.20	0.050
Metastatic LN	1.14 ± 2.04	1.38 ± 2.58	0.91 ± 1.38	0.455
Tumor diameter (cm)	1.05 ± 0.75	0.88 ± 0.60	1.21 ± 0.85	0.149
Pathologic diagnosis				1.000
PTC	34 (77.3%)	17 (81.0%)	17 (73.9%)	
HCC	1 (2.3%)	0	1(4.3%)	
Benign	9 (20.5%)	4 (19.0%)	5 (21.7%)	
ETE				1.000
Yes	1 (2.3%)	0	1 (4.3%)	
No	43 (97.7%)	21 (100.0%)	22 (95.7%)	
Thyroiditis				0.042
Yes	12 (27.3%)	9 (42.9%)	3 (13.0%)	
No	32 (72.7%)	12 (57.1%)	20 (87.0%)	
Pathological T stage *				1.000
1a	26 (74.3%)	13 (76.5%)	13 (72.2%)	
1b	8 (22.9%)	4 (23.5%)	4 (22.2%)	
3b	1 (2.9%)	0	1 (5.6%)	
Pathological N stage *				0.845
0	15 (42.9%)	7 (41.2%)	8 (44.4%)	
1a	20 (57.1%)	10 (58.8%)	10 (55.6%)	
AJCC (8th edition) stage *				1.000
I	33 (94.3%)	16 (94.1%)	17 (94.4%)	
II	2 (5.7%)	1 (5.9%)	1 (5.6%)	

* Available for the 35 patients with malignancy. Values are expressed as mean ± standard deviation or numbers (%). BMI, body mass index; CND, central node dissection; PTC, papillary thyroid cancer; HCC, Hürthle cell carcinoma; ETE, extrathyroidal extension.

**Table 2:** Group comparison of clinical outcomes.

		Group		
	Overall (*n* = 44)	Conventional (*n* = 21)	Mini-Flap (*n* = 23)	*p*-Value
Operating time (min)	191.92 ± 35.62	206.18 ± 31.09	178.90 ± 34.43	0.009
Flap-making time (min)	33.75 ± 8.12	38.85 ± 2.73	32.21 ± 8.62	0.003
Estimated blood loss (mL)	98.14 ± 48.89	92.05 ± 41.67	103.70 ± 55.00	0.436
Total drainage (mL)	172.61 ± 66.85	196.57 ± 81.40	150.74 ± 40.80	0.027
Hospital stay (days)	4.66 ± 1.18	4.86 ± 1.24	4.48 ± 1.12	0.293

Values are expressed as mean ± standard deviation or numbers (%).

**Table 3:** Group comparison of pain parameters.

	Group		
	Conventional (*n* = 21)	Mini-Flap (*n* = 23)	*p*-Value
Numeric rating scale score			
Postoperative 2 h	5.52 ± 0.87	4.57 ± 1.31	0.006
Postoperative 24 h	2.81 ± 0.40	3.00 ± 1.09	0.440
Postoperative 48 h	2.76 ± 0.44	2.87 ± 0.87	0.612
Postoperative 72 h	2.67 ± 0.48	2.39 ± 0.78	0.172
Number of analgesics			
Postoperative 2 h	0.95 ± 0.22	0.65 ± 0.49	0.012
Postoperative 24 h	0.29 ± 0.56	0.48 ± 0.59	0.276
Postoperative 48 h	0.48 ± 0.98	0.30 ± 0.77	0.518
Postoperative 72 h	0.00 ± 0.00	0.09 ± 0.29	0.162

Values are expressed as mean ± standard deviation or numbers (%).

**Table 4:** Group comparison of surgical outcomes.

		Group		
	Overall (*n* = 44)	Conventional (*n* = 21)	Mini-Flap (*n* = 23)	*p*-Value
Postoperative lowest iPTH (pg/mL)	38.81 ± 18.40	39.98 ± 17.89	37.75 ± 19.20	0.693
Postoperative hypoparathyroidism				1.000
No	41 (93.2%)	20 (95.2%)	21 (91.3%)	
Transient	3 (6.8%)	1 (4.8%)	2 (8.7%)	
Permanent	0	0	0	
Postoperative vocal cord palsy				0.599
No	41 (93.2%)	19 (90.5%)	22 (95.7%)	
Transient	3 (6.8%)	2 (9.5%)	1 (4.3%)	
Permanent	0	0	0	
Other complications	0	0	0	

Values are expressed as mean ± standard deviation or numbers (%). iPTH, intact parathyroid hormone.

**Table 5:** Group comparison of immediate-oncological outcomes.

		Group		
	Overall (*n* = 35)	Conventional (*n* = 17)	Mini-Flap (*n* = 18)	*p*-Value
Response to therapy				1.000
Excellent	31 (88.6%)	16 (94.1%)	15 (83.3%)	
Indeterminate	3 (8.6%)	1 (5.9%)	2 (11.1%)	
Biochemically incomplete	1 (2.9%)	0	1 (5.6%)	
Structurally incomplete	0	0	0	

Values are expressed as the mean ± standard deviation or numbers (%).

## Data Availability

The data presented in this study are available on request from the corresponding author.
